# The Endothelium and COVID-19: An Increasingly Clear Link Brief Title: Endotheliopathy in COVID-19

**DOI:** 10.3390/ijms23116196

**Published:** 2022-05-31

**Authors:** Isabelle Six, Nicolas Guillaume, Valentine Jacob, Romuald Mentaverri, Said Kamel, Agnès Boullier, Michel Slama

**Affiliations:** 1 UR 7517 UPJV, Pathophysiological Mechanisms and Consequences of Cardiovascular Calcifications (MP3CV), Picardie Jules Verne University, 80025 Amiens, France; romuald.mentaverri@chu-amiens.fr (R.M.); said.kamel@u-picardie.fr (S.K.); boullier.agnes@chu-amiens.fr (A.B.); slama.michel@chu-amiens.fr (M.S.); 2 EA Hematim 4666, Picardie Jules Verne University, 80025 Amiens, France; guillaume.nicolas@chu-amiens.fr (N.G.); jacob.valentine@chu-amiens.fr (V.J.); 3 Amiens-Picardie University Medical Center, Human Biology Center, 80054 Amiens, France; 4 Amiens-Picardie University Medical Center, Medical Intensive Care Unit, 80054 Amiens, France

**Keywords:** COVID-19, SARS-CoV-2, endothelium

## Abstract

The endothelium has a fundamental role in the cardiovascular complications of coronavirus disease 2019 (COVID-19). Infection with severe acute respiratory syndrome coronavirus 2 (SARS-CoV-2) particularly affects endothelial cells. The virus binds to the angiotensin-converting enzyme 2 (ACE-2) receptor (present on type 2 alveolar cells, bronchial epithelial cells, and endothelial cells), and induces a cytokine storm. The cytokines tumor necrosis factor alpha, interleukin-1 beta, and interleukin-6 have particular effects on endothelial cells—leading to endothelial dysfunction, endothelial cell death, changes in tight junctions, and vascular hyperpermeability. Under normal conditions, apoptotic endothelial cells are removed into the bloodstream. During COVID-19, however, endothelial cells are detached more rapidly, and do not regenerate as effectively as usual. The loss of the endothelium on the luminal surface abolishes all of the vascular responses mediated by the endothelium and nitric oxide production in particular, which results in greater contractility. Moreover, circulating endothelial cells infected with SARS-CoV-2 act as vectors for viral dissemination by forming clusters that migrate into the circulation and reach distant organs. The cell clusters and the endothelial dysfunction might contribute to the various thromboembolic pathologies observed in COVID-19 by inducing the formation of intravascular microthrombi, as well as by triggering disseminated intravascular coagulation. Here, we review the contributions of endotheliopathy and endothelial-cell-derived extracellular vesicles to the pathogenesis of COVID-19, and discuss therapeutic strategies that target the endothelium in patients with COVID-19.

## 1. Introduction

Although coronavirus disease 2019 (COVID-19) was initially considered to be a respiratory disease, it is associated with a number of cardiovascular complications. COVID-19 is caused by the highly pathogenic severe acute respiratory syndrome coronavirus 2 (SARS-CoV-2). The virus infects humans by binding to through the angiotensin-converting enzyme 2 (ACE-2) receptor expressed notably by type 2 alveolar cells, bronchial epithelial cells, and endothelial cells [1,2].

Endothelial cells have many functional properties, and are key regulators of coagulation, inflammation, oxidative stress, and vasomotricity. Viral diseases can damage the endothelium in many ways. Viruses can induce not only the apoptosis of endothelial cells, but also induce massive increases in cytokine levels, resulting in changes in cell junctions, elevated vascular permeability, and endothelial dysfunction.

A number of macro thromboembolic events (e.g., pulmonary embolism), micro thromboembolic events (e.g., microthrombosis in small pulmonary arteries), and disseminated intravascular coagulation have been described in patients with COVID-19; the endothelial cells’ roles in these phenomena have been described [3].

## 2. The Endothelium

### 2.1. Endothelial Cells

The intima (the innermost layer of the vessel, directly in contact with circulating blood) comprises a monolayer of endothelial cells. These cells are oriented parallel to the circulating flow. The cell–cell junctions are complex structures involved in the regulation of vascular permeability. Adherens junctions and tight junctions are found next to the connecting junctions that enable cell–cell exchanges [4]. The presence and relative density of these junctions varies along the vascular tree, which therefore modulates the endothelium’s permeability. The surface of endothelial cells in contact with the blood is covered with a glycocalyx, which prevents reactions with blood components such as platelets and leukocytes [5]. Endothelial cells proliferate more rapidly in vitro than in vivo: the doubling time is around 48 h in vitro, and between a month and a year in vivo in adults. Endothelial cells regulate many functions, and the endothelium has essential roles in vasomotricity, vascular permeability, and vascular homeostasis.

### 2.2. Endothelial Dysfunction 

**Figure 1:**
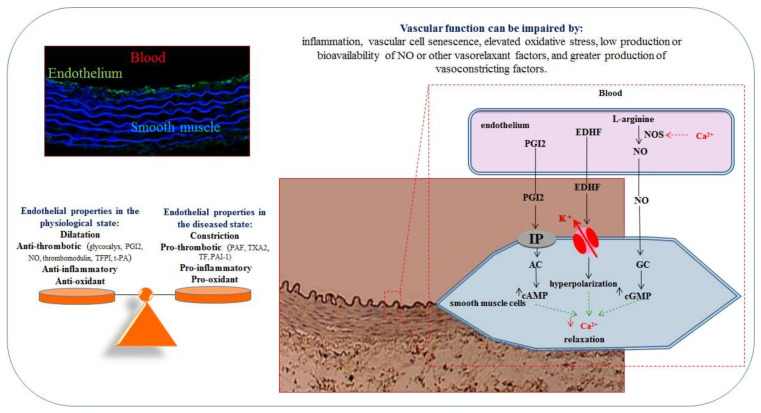
The endothelium influences the blood vessel’s health. PGI2, prostacyclin; TXA2, thromboxane A2; EDHF, endothelium-derived hyperpolarizing factor; NO, nitric oxide; NOS, nitric oxide synthase; IP, prostacyclin receptor; AC, adenylate cyclase; GC, guanylate cyclase; cAMP, cyclic adenosine monophosphate; cGMP, cyclic guanosine monophosphate; TFPI, tissue factor pathway inhibitor; t-PA, tissue plasminogen activator; PAF, platelet-activating factor; TF, tissue factor; PAI-1, plasminogen activator inhibitor-1.

The endothelium influences the vessel’s health (Figure 1). Under normal circumstances, the endothelium has anticoagulant, anti-inflammatory, and anti-oxidant properties. However, in a pathological situation, the endothelium acquires prothrombotic, pro-inflammatory, and pro-oxidant properties. The balance is very fragile, and the transition from a functional endothelium to a dysfunctional endothelium state is very rapid, and takes time to be reversed. 

The endothelium also forms a large surface area that regulates hemodynamic functions by secreting relaxing and contracting factors. Vascular dysfunction is characterized by a breakdown in the balance between the production of relaxing vs. contracting factors. The mechanisms underlying this impairment of vascular function include (i) inflammation, (ii) the senescence of vascular cells, (iii) the increase in oxidative stress, (iv) low production or release of nitric oxide (NO) or other relaxing factors, and (v) the elevated production of vessel-contracting factors.

In many cardiovascular diseases, endothelial dysfunction is characterized by impaired relaxation and a decrease in the amount or potency of NO. The production of NO from the terminal guanidine group of L-arginine is catalyzed by nitric oxide synthase (NOS). A decrease in the amount of NO may be due to (i) a decrease in the amount of substrate (i.e., L-arginine), (ii) a decrease in the activity of endothelial NOS (eNOS), or (iii) an increase in circulating levels of asymmetric dimethylarginine (a natural analogue of L-arginine that acts as an endogenous NOS inhibitor) [6]. A loss of potency may be due to a reduction in the NO sensitivity of guanylate cyclase in vascular smooth muscle cells [7].

Oxidative stress resulting from the overproduction of reactive oxygen species (ROS) reduces vessel relaxation, and potentiates vessel contraction. Superoxide anions (O2°^−^) form peroxynitrites by interacting with NO, and thus decrease the latter’s bioavailability. Moreover, ROS decrease NO’s effects by reducing guanylate cyclase expression and sensitivity [8,9]. The presence of excess ROS sets up a vicious circle by inhibiting antioxidant systems and decoupling eNOS, which then becomes a source of superoxide anions [10,11,12]. Furthermore, ROS decrease the activity of calcium-sensitive potassium channels, and decrease vasorelaxation induced by endothelium-derived hyperpolarizing factor (EDHF) [13,14]. ROS potentiate vascular contraction by decreasing NO’s downregulation of endothelin 1 production and increasing levels of endoperoxides, prostaglandins [15], and intracellular calcium [16]. Lastly, ROS activate cyclooxygenases and induce the formation of isoprostanes, which bind to thromboxane A2 receptors and induce powerful vessel contraction [17,18,19].

Vascular function can be impaired by abnormally high levels of vasoconstrictors, such as angiotensin. Within the renin angiotensin system, angiotensinogen (produced mainly in the liver) is transformed by renin into angiotensin I, which, in turn, is transformed by angiotensin-converting enzyme into angiotensin II (Ang II). The latter then binds to angiotensin type 1 (AT1) receptors, leading to the release of endothelin 1. Lastly, endothelin 1 binds to its cognate receptors on smooth muscle cells, activates a signaling pathway, and thus induces smooth muscle contraction.

Chronic or acute exposure to harmful circulating agents (such as viruses, uremic toxins, and bacteria) alters the properties of the endothelium, which becomes activated and dysfunctional [20]. Endothelial activation leads to the detachment of endothelial cells into the circulation and/or the release of endothelial extracellular vesicles (EVs).

### 2.3. Endothelial Extracellular Vesicles

EVs constitute a heterogenous group of membrane-derived particles with a lipid bilayer. They include exosomes, microvesicles, and apoptotic bodies, which are released from the cell in response to activation or apoptosis. Exosomes are released continuously from cells, whereas microvesicles and apoptotic bodies are released predominantly by activated or apoptotic cells. EVs are generated by plasma membrane blebbing, which induces the externalization of phosphatidylserine, normally located exclusively in the cytoplasmic leaflet [21].

Along with direct cell–cell contact and the release of secreted molecules, EVs are involved in cell–cell communication. By transporting and delivering various bioactive cargos (such as proteins, lipids, mRNAs, micro RNAs (miRNAs), and DNA), Evs exert positive, neutral, or detrimental effects on the recipient cells by modulating gene expression, influencing the cell phenotype, affecting molecular pathways, and mediating biological behaviors [22].

Most of the EVs in the blood originate from platelets and erythrocytes. The proportion of circulating EVs secreted by endothelial cells is relatively low under physiological conditions, but is markedly greater under pathological conditions with endothelial dysfunction. EVs released by endothelial cells contain many endothelial markers, such as endoglin (CD105), E-selectin (CD62E), S-endo (CD146), vascular endothelial cadherin (CD144), platelet endothelial cell adhesion molecule 1 (PECAM1, CD31), and intercellular adhesion molecule 1 (ICAM-1, CD54) [23]. Many of the stimuli leading to endothelial cell activation are associated with a greater production of EVs; these stimuli include a decrease in shear stress [24,25], hypoxic conditions [26], and inflammatory conditions [27]. In a mouse model, EVs derived from activated endothelial cells (after treatment with a high glucose concentration) amplified endothelial inflammation, and impaired endothelial function by promoting activation of the endothelium via an increase in NADPH oxidase activity [28]. Thereby, endothelial EVs are not only useful as biomarkers of endothelial injury, but can also participate in the progression of cardiovascular disease. For instance, the miRNA cargo of EVs derived from endothelial cells in an inflammatory context has been linked to greater expression of vascular endothelial growth factor B in the recipient cerebrovascular pericytes [29].

Moreover, proteomic analyses have revealed the presence of a full set of antioxidant molecules enclosed in endothelial cell-derived EVs, including glutathione transferase, glutathione peroxidases, peroxiredoxins, and thioredoxins [30], all of which protect against oxidative stress-induced cell death [31].

In some circumstances, EVs released from blood cells can have harmful effects on endothelial cells; the vesicles trigger oxidative damage and cell death by reducing NO release in the endothelial cells. For example, EVs from patients with sepsis have high levels of NADPH oxidase activity; this generates ROS and triggers apoptosis in endothelial cells [32].

## 3. Endothelial Cells and Viruses

SARS-CoV-2 is part of the coronavirus family, along with severe acute respiratory syndrome coronavirus 1 (SARS-CoV-1) and Middle East respiratory syndrome coronavirus (MERS-CoV)—both of which can also cause a potentially lethal disease. SARS-CoV-1 is mainly a lower respiratory tract disease that results in pulmonary damage and respiratory distress [33]. As in COVID-19, the extrapulmonary manifestations of SARS-CoV-1 infection include systemic vasculitis, and the swelling and apoptosis of endothelial cells. Endothelial cells express high levels of ACE2, but are not infected by SARS-CoV-1 [34]. Hence, the vascular abnormalities and inflammatory changes in various organs associated with SARS-CoV-1 infection might therefore be due to the systemic toxic effects of the immune response to the virus.

Although some of the viruses replicate productively in endothelial cells, many of the associated features of the disease (including impairments of the vascular system) are thought to result from the infected cells’ release of mediators.

SARS-CoV-2, SARS-CoV-1, and MERS-CoV all cause an excessive host response, in which a cytokine storm induces severe disease and, in some cases, death [35,36,37]. In most moribund patients, SARS-CoV-2 infection is characterized by elevated plasma concentrations of interleukins (ILs)-1β, 2, 6, 7, and 10, granulocyte-colony stimulating factor, interferon-γ-inducible protein 10, monocyte chemoattractant protein 1, macrophage inflammatory protein 1 alpha, and tumor necrosis factor alpha (TNF-α) [35,36,37].

The cytokines TNF-α, IL-6, and IL-1β have particular effects on endothelial cells. TNF-α affects the regulation of vascular permeability by directly modifying tight junction composition and structure through signaling pathways that are independent of cell death [38]. IL-6 treatment in vitro also increases permeability across endothelial cell layers, and results in tight junction mislocalization, actin structure remodeling, and increased actin contractility [39]. Lastly, IL-1β has been found to suppress endothelial cell proliferation [40].

Many studies have highlighted the effects of cytokines on vascular function. As early as 1989, Aoki et al. reported that TNF-α reduced endothelium-dependent relaxation, and suggested that cytokine activity was associated with endothelial vasodilator dysfunction [41]. Vascular dysfunction is mainly due to the decreased secretion or accelerated catabolism of relaxing factors such as NO, together with (in some cases) a greater production of contractile factors. TNF-α has two main effects on vascular reactivity: it decreases vasorelaxation, and induces the contraction of arterial segments. High TNF-α concentrations directly decrease levels of eNOS, which shortens the half-life of NO [42]. It is noteworthy that TNF-α reduces degradation of asymmetric dimethylarginine, which acts on endothelial cells by accelerating senescence and inducing oxidative stress [43]. TNF-α is also able to promote the production of oxygen-derived free radicals in endothelial cells [44], which contributes to the cytokine’s pro-contraction properties.

IL-6 modulates vascular function by upregulating the mRNA expression of the angiotensin II type 1 receptor, which then leads to greater angiotensin II-mediated vasoconstriction, greater free oxygen radical production (in vitro and in vivo), and the onset of endothelial dysfunction [45].

These data suggest that endothelial cells are major targets for cytokine signals. Moreover, exposure of the endothelium to cytokines modulates vascular inflammation, accelerates oxidative stress and endothelial cell apoptosis, and contributes to endothelial dysfunction.

## 4. The Endothelium and COVID-19

Various studies have demonstrated that SARS-CoV-2 can infect endothelial cells [3], and that it induces the secretion of pro-inflammatory cytokines [35].

Although the direct effects of SARS-CoV-2 on endothelial cells and the consequences on cell apoptosis and function have not been fully characterized, the intracellular presence of viral bodies confirms the virus’s involvement [3]. Moreover, alterations in endothelial barrier function and vascular permeability have been suspected.

It is now increasingly clear that COVID-19 is not solely a respiratory disease, and that it can lead to cardiovascular complications. One study revealed the presence of viral elements inside endothelial cells, an accumulation of inflammatory cells, and evidence of endothelial and inflammatory cell death [3].

**Figure 2:**
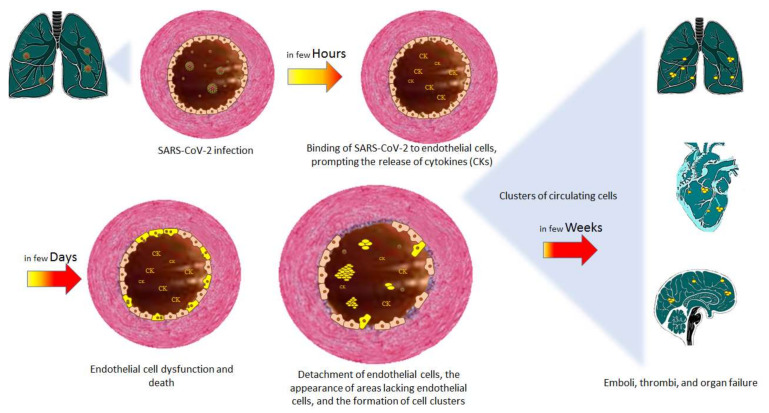
The time course of SARS-CoV-2 infection, the consequences for endothelial cells, and the resulting pathologies.

In view of the factors described above and the literature data on SARS-CoV-2’s mechanism of action, one can hypothesize that the time course of SARS-CoV-2’s effects on the endothelium is as shown in Figure 2.

In the lung, SARS-CoV-2 binds to the ACE-2 receptor on the endothelial cells’ surface. The intracellular presence of viral bodies [3] confirms the virus’s involvement—probably via the induction of apoptosis and pyroptosis.

The cytokine storm activates the endothelium, leading to endothelial dysfunction, endothelial cell death, changes in tight junctions, vascular hyperpermeability, and endothelial barrier dysfunction.

The endothelial dysfunction, the apoptosis and pyroptosis of endothelial cells, and the changes in tight junction all contribute to cell detachment. Endothelial cell-free areas start to appear on the intimal surface, and the detached endothelial cells pass into the circulation. It has been shown that both adherent and detached apoptotic endothelial cells become procoagulant via the elevated expression of phosphatidylserine and the loss of anticoagulant components, such as thrombomodulin and tissue factor (TF) pathway inhibitor [46]. This effect is associated with an elevation of TF production, which activates secondary hemostasis and induces elevated levels of chemokines and pro-inflammatory cytokines that contribute to the inflammatory response observed during COVID-19. The appearance of TF in the bloodstream initiates the extrinsic coagulation cascade, which leads to disseminated intravascular coagulation (characterized by the generalized, uncontrolled activation of coagulation). The main abnormalities in hemostasis are elevated D-dimer and fibrinogen levels, moderate thrombocytopenia, low prothrombin levels, and the lengthening of the activated partial thromboplastin time [47]. In patients infected with SARS-CoV-2, elevated D-dimer levels are prognostic markers for the development of severe COVID-19 requiring intensive care or resulting in death [47,48].

Under normal conditions, apoptotic cells are removed in the blood stream, and are replaced rapidly by regenerated endothelial cells. In COVID-19, the detachment of endothelial cells can take place very quickly, and endothelial cells might not be regenerated as effectively as usual.

The detachment of endothelial cells induces a number of phenomena. The circulating SARS-CoV-2-infected endothelial cells can form clusters, migrate through the circulation, reach distant organs, disseminate the virus, and thus induce various pathologies observed in COVID-19 (e.g., lung, cardiovascular, and neurologic diseases). Loss of endothelium on the luminal surface also abolishes the vascular reactivity mechanisms mediated by the endothelium in general, and NO production in particular. The loss of NO production results in a lower vessel relaxation capacity and a marked increase in vessel contraction. NO not only prevents abnormal contraction, but also inhibits the aggregation of platelets, the expression of adhesion molecules at the surface of the endothelial cells, and hence the adhesion and penetration of white blood cells. The decrease in NO production abolishes all of the mediator’s protective effects on coagulation and inflammation. Moreover, disruption of the endothelial barrier enables aggregating platelets to approach vascular smooth muscle cells and trigger a contraction of the latter via the release of thromboxane A2 and serotonin, which initiates the vascular phase of hemostasis. The detachment of endothelial cells from the luminal surface and the abolition of the vessel relaxation induce the formation of the intravascular microthrombi responsible for the severe consequences of COVID-19. Hence, the disease rapidly attacks endothelial cells, regardless of the mechanism involved.

### 4.1. Endothelial Dysfunction in COVID-19

The existence of COVID-19-associated endotheliopathy was recognized very soon after the emergence of the disease; autopsy studies revealed the direct infection of endothelial cells by SARS-CoV-2 [3,49]. Patients with COVID-19 had elevated levels of endothelial dysfunction biomarkers. Circulating levels of markers of endothelial injury (e.g., von Willebrand factor (vWF), soluble thrombomodulin, angiopoietin 2, and follistatin) were higher in hospitalized patients with COVID-19 than in healthy controls, and elevated thrombomodulin, angiopoietin 2, and follistatin levels were correlated with higher mortality rates in patients admitted to the intensive care unit (ICU) [50,51]. The COVID-19-associated endotheliopathy contributes to a hyperinflammatory response, hypercoagulability, and thus thrombosis events.

The endothelial dysfunction in COVID-19 was evaluated recently in six patients with a laboratory-confirmed SARS-CoV-2 infection. Overall, four patients were positive for endothelial dysfunction, with reactive hyperemia index values ranging between 1.13 and 1.56 (mean value: 1.32; normal values: >1.67) [52]. These findings confirmed that patients with COVID-19 are at a greater risk of developing endothelial dysfunction, and that endothelial impairment can occur even in the absence of cardiovascular risk factors.

### 4.2. Endothelial Extracellular Vesicles in COVID-19

Several markers of vascular endothelial cells and glycocalyx activation have been characterized in patients with COVID-19 requiring ICU admission [53]. Compared with healthy controls, these patients had higher vWF, chondroitin sulfate, and syndecan levels. Moreover, endothelial dysfunction is known to be associated with the generation of EVs. High numbers of large EVs (100–300 nm) and small EVs (30–100 nm) are found in plasma samples from patients with COVID-19. Analysis of the protein cargo in the large EVs revealed various profiles related to disease severity, including specific changes in prothrombotic factors in particular [54]. The circulating EVs also expressed TF, the levels of which were correlated with the prothrombin time and levels of fibrinogen, vWF, and a disintegrin and metalloprotease with thrombospondin type I repeats-13. Levels of EV-associated TF activity were higher in patients with COVID-19 than in controls, and were correlated with disease severity (e.g., oxygen requirement and death). The EV type was also strongly correlated with D-dimer levels. These data suggest that EVs have a role in thrombosis in patients with COVID-19 [55,56].

Endothelial Evs and oxidative stress are closely associated. Firstly, oxidative stress increases the production of endothelial-cell-derived EVs, and modifies their contents [57,58]. Secondly, endothelial-cell-derived EVs produce ROS that can be delivered to neighboring cells, and thus spread oxidative damage. Brodsky et al. showed that endothelial EVs inducs O_2_^−^ production in cultured renal microvascular endothelial cells, as well as in ex vivo aortic rings [59].

Many studies have shown that endothelial-derived EVs can influence endothelial cell function. There appears to be a reciprocal relationship between EVs and NO-dependent endothelial dysfunction. Indeed, NO downregulates EV production [60], and, under certain conditions, eNOS decoupling can stimulate the production of EVs [61]. Endothelial cell-derived EVs might impair endothelium-dependent vasorelaxation in a paracrine manner. It has been reported that EVs reduce endothelial NO bioavailability by decreasing eNOS phosphorylation [59,62,63,64] and activity [65], or by inducing oxidative stress [60]. Moreover, EVs contain NADPH oxidase, an enzyme that synthetizes ROS, and the superoxide anion in particular [66,67]; the latter could rapidly react with NO to form peroxynitrite, a reactive nitrogen species.

Endothelial-derived EVs can also have beneficial effects by scavenging ROS. Bodega et al. showed that cultured endothelial cells produce EVs that possess antioxidant machinery, and that can scavenge ROS [30,68]. In fact, EVs can transport antioxidant molecules to target cells, which internalize the cargo and are thus better protected against oxidative stress [69]. Similarly, Mahmoud et al. showed that endothelial microparticles prevent lipid-induced endothelial damage via Akt/eNOS signaling and reduced oxidative stress [64].

All of these factors might explain the endothelial dysfunction observed in patients with COVID-19, since elevated levels of oxidative stress and reactive nitrogen species, as well as elevated EV counts, have been reported in acute cases. The EVs released during COVID-19 might also lead to a decrease in NO bioavailability, which in turn would induce the endothelial dysfunction observed in patients with COVID-19.

Given the huge impact of the COVID-19 pandemic worldwide, the medical and scientific communities have worked together to develop ways of limiting the consequences of this viral infection.

## 5. Therapeutic Management of Patients with COVID-19

Patients with a positive lateral flow test or PCR test for SARS-CoV-2, a risk of a severe form of the disease (aged 65 or over and/or with comorbidities), and who are immunocompromised or not fully vaccinated should consult a physician for initial management and the initiation of follow-up. The clinical examination includes an oxygen saturation measurement, which conditions the choice of ambulatory management (SpO_2_ ≥ 95%). If there are risk factors for severe COVID-19, the physician may prescribe the use of a pulse oximeter for enhanced home monitoring [70].

If the patient’s condition deteriorates, treatment is required. In France, the management of patients with COVID-19 was specified in a report by the High Council for Public Health (*Haut Conseil de Santé Publique*, 28 January 2021); it is based on supportive and preventive treatments, including appropriate oxygen therapy, analgesics, antipyretics, thrombotic risk prevention, and antibiotics in suspected cases of bacterial co-infection.

As of 20 March 2022, four therapeutics have been fast-tracked for the management of people at a high risk of severe COVID-19: Paxlovid^®^, Evusheld^®^, Ronapreve^®^, and Xevudy^®^. All of these therapeutics are being closely monitored with regard to their safety and effectiveness—particularly in light of the emergence of new variants of SARS-CoV-2.

### 5.1. Antiviral Compounds

Paxlovid^®^ (PF-07321332–ritonavir) is indicated for the treatment of COVID-19 in adults who do not require oxygen therapy, and who are at high risk of progression to severe COVID-19. PF-07321332 is a peptidomimetic inhibitor of the coronavirus 3C-like protease, and ritonavir inhibits CYP3A-mediated metabolism of PF-07321332 (resulting in increased plasma concentrations of PF-07321332). Paxlovid should be administered as soon as possible after the diagnosis, and within five days of the onset of symptoms.

Veklury^®^ (remdesivir) is indicated in the treatment of adults and adolescents (aged 12 or over, and weighing at least 40 kg) with COVID-19 and receiving low-flow or high-flow oxygen therapy for pneumonia or non-invasive or invasive assisted ventilation or extracorporeal membrane oxygen therapy.

### 5.2. Anti-SARS-CoV-2 Monoclonal Antibodies 

Like proposed in Table 1, Evusheld^®^ (tixagevimab + cilgavimab) is indicated for pre-exposure prophylaxis of COVID-19 in adult and adolescent patients (aged 12 or over and weighing at least 40 kg) with disease- or treatment-related immunodeficiencies, who are not fully vaccinated, non-responders to vaccination, or are ineligible for vaccination, and have a high risk of severe COVID-19. 

Ronapreve^®^ (casirivimab + imdevimab for intravenous infusion) is indicated in the treatment of hospitalized SARS-CoV-2-positive patients aged 12 or over who do not require oxygen therapy. Treatment with Ronapreve^®^ should be initiated as soon as possible after a positive PCR test for SARS-CoV-2, and within five days of symptom onset. Casirivimab and imdevimab are both human monoclonal IgG1 antibodies. This combination is also indicated for post-exposure prophylaxis of SARS-CoV-2 infections in patients (aged 12 or over) who have not developed a response to a complete vaccination regimen, due to immunosuppression.

Xevudy^®^ (sotrovimab) is indicated in the treatment of adults and adolescents (aged 12 or over, and weighing at least 40 kg) with COVID-19, who do not require oxygen supplementation and who are at risk of progressing to a severe form of the disease (as defined by the French Research Agency for Emerging Infectious Diseases). Sotrovimab is a novel human monoclonal IgG1 that binds to a highly conserved epitope of the SARS-CoV-2 spike protein’s receptor binding domain. However, sotrovimab fails to neutralize the BA.2 variant of SARS-CoV-2, and is no longer being recommended for use in hospitals.

Roactemra^®^ (tocilizumab, an anti-IL6 antibody) has received specific regulatory approval for the treatment of COVID-19 in adults receiving systemic corticosteroid therapy and requiring oxygen supplementation or mechanical ventilation. Tocilizumab can be used in patients requiring high-flow oxygen therapy, displaying marked inflammation (C-reactive protein ≥ 75 mg/L), and who fail to improve after 48 h of standard care, including dexamethasone (or an equivalent corticosteroid). In contrast, the use of tocilizumab is not recommended in patients on invasive mechanical ventilation. In March 2022, sarilumab (anti-IL6) and anakinra (anti-IL1) were no longer recommended in any situation. There is a lack of data in immunocompromised patients (a population at a high risk of severe COVID-19), since the likelihood of a concomitant non-SARS-CoV-2-related infection may be increased by the use of this immunosuppressant.

Despite the endothelium’s proven role in the pathophysiology of COVID-19, none of the above-mentioned therapeutics target the maintenance or restoration of normal vascular function.

## 6. Treatments That Target Endothelial Dysfunction

Several currently marketed drugs are potentially effective in restoring vascular function. Given the causal role of NO pathways and oxidative stress in vascular alterations, molecules targeting these signaling pathways have been developed. The therapeutic efficacy of vasodilators such as prostacyclin have also been evaluated.

Drugs targeting the NO pathway (such as nitrate derivatives and NO donors) influence the production of this mediator or its mechanism of action. Although the topic remains subject to debate, the literature data suggest that supplementation with or intra-arterial infusion of L-arginine is associated with an increase in the vessel’s functional capacities [71,72,73,74]. Similarly, the use of type 3 phosphodiesterase inhibitors, such as cilostazol, improved vascular function and reduced symptoms in patients with peripheral arterial disease [75].

The best-known antioxidants include ß-carotene (provitamin A), ascorbic acid (vitamin C), tocopherol (vitamin E), polyphenols (resveratrol), lycopene, glutathione, and enzymes such as catalase, superoxide dismutase, and some peroxidases. Antioxidants found in the diet include flavonoids (e.g., quercetin, a widespread natural product in plants), tannins (found in cocoa, coffee, tea, grapes, etc.), anthocyanins (in red fruits), and phenolic acids (in cereals, fruits, and vegetables).

The antioxidant properties of ascorbic acid (vitamin C, 1000 mg/day) and alpha-tocopherol (vitamin E, 1000 IU/day) result in a relative decrease in superoxide anion generation rate (10 ± 4 nmol/min/g with vitamin C, 9.6 ± 3.5 nmol/min/g with vitamin E, and 21 ± 9 nmol/min/g with controls; *p* < 0.05). These effects are associated with lower activation of vascular NADPH oxidase, and greater activation of superoxide dismutase. With regard to vascular function, treatment with vitamin C or E improves acetylcholine-induced vasodilation [76].

The literature data concerning the effects of antioxidants on vascular function are subject to debate. Ashor et al. reviewed this topic by searching the MEDLINE, Embase, Cochrane Library, and Scopus databases from their creation up until May 2014. The review covered 46 randomized, controlled trials, with a total of 1817 adult participants having received vitamin C alone, vitamin E alone, or a combination of them both for more than 2 weeks. The investigators concluded that significant improvements in vascular function were observed when administering vitamin C alone (500 to 2000 mg/day) or vitamin E alone (300 to 1800 IU/day), whereas co-administration of both treatments was ineffective [77].

Applying the same approach to the effects of vitamin D on vascular function, an analysis of 1177 patients in 16 different trials did not evidence a significant overall effect on vascular function. Nevertheless, a subgroup analysis highlighted a significant improvement in endothelial function in diabetic subjects receiving vitamin D supplementation [78].

Many antihypertensive drugs are associated with improved vascular function. The effects of angiotensin-converting enzyme inhibitors (ACEIs) on vascular function are particularly interesting, and the role of angiotensin II receptor blockers (ARBs) has been clearly demonstrated.

ACEIs have several effects on the vascular system. Firstly, they inhibit vasoconstriction, produce an antioxidant effect, and limit the production of vasoconstrictor substances, such as endothelin 1. Secondly, the inhibition of bradykinin degradation by ACEIs stimulates bradykinin receptors and thus promotes NO production. Thirdly, the thiol group present in some ACEIs (e.g., zofenopril and enalapril) provides direct antioxidant activity.

The clinical benefits of ACEI-associated improvements in endothelial function have been widely demonstrated [79,80,81,82,83,84]. For example, Mancini et al. studied the effects of quinapril vs. placebo in patients with coronary heart disease, but who were free of heart failure, cardiomyopathy or major lipid abnormalities; these criteria minimized the effects of these variables on endothelial dysfunction. After 6 months of treatment, vascular function was significatively better in the quinapril group than in the placebo group (mean ± standard deviation increase in the coronary artery diameter response to incremental acetylcholine concentrations: 12.1 ± 3.0% vs. −0.8 ± 2.9%, respectively; *p* < 0.002) [85].

The adrenal and vascular effects of Ang II are mainly exerted through AT1 receptors. Ang II is a very potent vasoconstrictor, and ARBs are competitive inhibitors of the Ang II–AT1 receptor interaction. Early studies of the effects of ARBs on endothelial function showed that the chronic administration of losartan or irbesartan restored relaxation in patients with hypertension [86,87]. The ability of other ARBs (such as valsartan) to restore endothelial function are subject to debate; however, several studies have shown that treatment with valsartan for at least one year is associated with better endothelial function and lower levels of oxidative stress [88,89]. A study of 31 hypertensive patients compared the respective endothelial function effects of olmesartan and the calcium channel blocker amlodipine. Although the blood pressure fell to a similar extent in the two treatment groups, endothelial function was significantly improved in the olmesartan group only—possibly as a result of the compound’s antioxidant properties [90].

## 7. COVID-19 Treatments That Target the Endothelium or Endothelial Extracellular Vesicles 

Our current understanding of the mechanisms of COVID-19 endotheliopathy has prompted thoughts of therapies that reduce endothelial cell apoptosis or improve endothelial function like described in Table 2.

SARS-CoV-2’s tropism for endothelial cells leads to cell apoptosis, and thus a decrease in endothelial NO production [3,91]. Inhaled NO might be a therapeutic option in COVID-19 [92]. Inhaled NO has proven pulmonary vasodilation activity. Early administration of inhaled NO might be a safe, effective approach in the prevention of the development of severe forms of COVID-19. The beneficial effect of inhaled NO on vascular function might be associated with antiviral activity in patients with COVID-19. In vitro experiments have demonstrated that NO has antiviral activity against SARS-CoV-2 [93]. Although the modalities of inhaled NO administration (particularly the timing) still need to be explored, early initiation of this treatment might be promising [92].

It is also possible to enhance NO’s vasodilating effects by administering phosphodiesterase inhibitors. The most important drug used to restore vasodilatation is the selective phosphodiesterase-3 inhibitor cilostazol, which also has antiplatelet activity. Cilostazol’s potential activity has been studied in silico. Cilostazol can bind to the SARS-CoV-2’s main protease, as well as to the spike protein [94,95]. Despite cilostazol’s likely clinical potential [96], there are no published data on beneficial effects in patients with COVID-19.

The hormone prostacyclin is produced and secreted by endothelial cells; it induces vasodilatation, and acts as a platelet inhibitor [97]. Moreover, prostacyclin protects the endothelium and the endothelial glycocalyx, and has anti-inflammatory effects [98,99,100,101,102,103,104,105]. The prostacyclin analogue iloprost has shown beneficial effects in patients with COVID-19 through its ability to protect the endothelium and its antithrombotic activity [106]. In adults with COVID-19 requiring mechanical ventilation and who had a plasma thrombomodulin level >4 ng/mL, a 72 h infusion of prostacyclin (1 ng/kg/min) was associated with a longer median 28-day survival time without mechanical ventilation (16 days vs. 5 days in the placebo group). Moreover, the 28-day mortality rate and the 7-day incidence of serious adverse events were lower (by 50% and 81%, respectively) in the prostacyclin group than in the placebo group [107]. In a post-hoc study, the same researchers found that prostacyclin infusion resulted in less endothelial glycocalyx shedding. The plasma syndecan-1 level at 24 h had fallen by 3.95 ng/mL in the prostacyclin group and had risen by 3.06 ng/mL in the placebo group; this suggested a protective effect on the endothelium, which might have been related to the observed reduction in organ failure [108].

Potentially beneficial effects of other agents (e.g., antioxidants, such as vitamins C and D) on the endothelium in COVID-19 have been explored. It was not clear whether vitamin C provided any benefit in this setting. Hence, Gavrielatou et al. performed an observational cohort study and a meta-analysis of data from 1,807 patients. The researchers found that vitamin C administration was not associated with lower mortality among critically ill patients with COVID-19 [109].

Concerning the administration of vitamin D, Murai et al. reported on the administration of a single oral dose of vitamin D 200,000 IU (or placebo) in a cohort from 240 hospitalized patients in Brazil [110]. There was no difference in the hospital length of stay between the vitamin D group and the placebo group. Moreover, the researchers found no intergroup differences in the secondary outcomes (mortality, ICU admission, and mechanical ventilation). Similarly, a meta-analysis did not find any conclusive evidence to show that vitamin D supplementation is associated with lower mortality, invasive ventilation, or ICU admission rates [111].

Due to SARS-CoV-2’s mechanism of infection via the ACE-2 receptor, one of the scientific community’s primary concerns has been the impact of RAS drugs on COVID-19 morbidity and mortality rates. Recently, two literature reviews highlighted the body of evidence showing that ACEIs and ARBs are not associated with an elevated risk of infection, greater disease severity, or a worse prognosis [112,113]. Moreover, recently published preclinical and clinical data indicate that ACEIs and ARBs are associated with a better COVID-19 outcome by reducing inflammatory responses and by triggering mechanisms that counteract viral entry. However, this hypothesis require further support, and strategies that directly activate RAS anti-inflammatory components (such as soluble ACE2, angiotensin 1–7 analogues, and Mas or AT2 receptor agonists) are now emerging [113].

There are not yet any specific treatments for COVID-19. Conventional, nonspecific treatments include infection prevention, supportive care (e.g., oxygen supplementation), and mechanical ventilation support. Given the importance of the interaction between SARS-CoV-2 and its entry receptor, ACE2, on host cells, recent studies have focused on novel approaches for blocking or impeding this mechanism. Monteil et al. and others have shown that soluble recombinant ACE2 (rhACE2) neutralizes SARS-CoV-2 by binding the spike glycoprotein, thereby reducing viral entry into Vero-E6 cells and engineered human organoids [114,115]. Several other studies have used neutralizing antibodies against specific epitopes on the SARS-CoV-2 spike glycoprotein to inhibit viral infection [116,117,118,119]. However, the latter strategy has limitations, since RNA viruses can mutate and therefore escape the antibody’s effects.

Recent studies have highlighted the role of EVs in COVID-19, as well as their therapeutic potential against virus infection. Indeed, plasma and serum from patients convalescing from COVID-19 have been used to combat active SARS-CoV-2 infections, which suggests the presence of unknown antiviral components. El Shennawy et al. sought to identify these antiviral components, and detected elevated circulating levels of ACE2-positive extracellular vesicles in the plasma of patients with COVID-19. Such high levels are usually associated with a pathogenic situation. In fact, ACE2-positive EVs appear to be part of an innate antiviral mechanism that acts as a decoy to protect host cells from SARS-CoV-2 infection. El Shennawy et al. showed that ACE2-positive EVs isolated from cells transduced with an ACE2 lentivirus were able to protect human ACE2 transgenic mice from SARS-CoV-2 infection by competing with cellular ACE2. The researchers estimated that up to 20 to 40 ACE2 molecules were present on each EV; this might explain the 80-fold higher efficacy in blocking viral infections, relative to rhACE2 [120].

EVs can be used as natural drug delivery system due to their involvement in cell–cell communication [121]. Thanks to their small size and low immunogenicity, EVs are able to transfer endogenous and exogenous drug compounds into recipient cells. To this end, donor cells can be genetically engineered so that a specific ligand is expressed on the released EVs; hence, the use of engineered, ACE2-positive EVs is attracting interest, and appears to be a promising strategy for countering SARS-CoV-2 infections. Recent studies have shown that ACE2-containing EVs bind to the SARS-CoV-2 spike protein and inhibit viral infection [122,123]. Furthermore, Xie et al. showed that EVs enriched with palmitoylated ACE2 can be used to treat COVID-19 [124]. After SARS-CoV-2 has interacted with ACE2, the spike protein’s subunit S2 is processed by the transmembrane serine protease 2. In an in vitro study of epithelial cell lines, Cocozza et al. demonstrated that EVs containing ACE2 alone or combined with transmembrane serine protease 2 blocked SARS-CoV-2 spike-dependent infection more effectively than soluble ACE2 [123]. Most recently, Scott et al. demonstrated that tetraspanin (CD63)-expressing EVs accentuate the effect of neutralizing antibodies, and can be used to target and inhibit SARS-CoV-2 [125].

It is noteworthy that no EV-based treatments have yet been approved by the health authorities. To the best of our knowledge, the effects of these treatments on endothelial function have not been studied in vitro. This type of study would be of great interest, since prevention of the virus-host cell interaction might rescue endothelial function.

Taken as a whole, these data suggest that EVs are promising therapeutic tools against SARS-CoV-2 infections. However some important issues need to be addressed for clinical use, such as the culture conditions, EV purification/quantification, and choice of the donor cells.

## 8. Conclusions

The endothelium has been implicated in COVID-19. SARS-CoV-2 can act on endothelial cells directly by binding to the ACE-2 receptor, and indirectly by inducing a cytokine storm. Endothelial cells form a very fragile monolayer on the vessel’s intimal surface. The detachment of infected endothelial cells might disseminate the virus through the circulation, since clusters of cells reach distant organs. These cell clusters might also be involved in the various thromboembolic events observed in COVID-19. A better understanding of the mechanism whereby SARS-CoV-2 virus causes endothelial cell injury and the latter’s consequences on COVID-19 outcomes might enable the development of novel therapeutic strategies based on endothelial stabilization.

## Figures and Tables

**Table 1:** Recommendations for the use of therapeutic monoclonal antibodies available in France.

	Pre-Exposure Prophylaxis	Post-Exposure Prophylaxis	Curative Treatment at Home	Curative Treatment in Hospital
Evusheld^®^ (tixagevimab + cilgavimab)	Yes	No	No	No
Ronapreve^®^ (casirivimab + indevimab)	No	Yes (delta)	Yes (delta)	Yes (delta)
Xevudy^®^ (sotrovimab)	No	No	No	Yes

**Table 2:** The effects of therapeutic approaches that target endothelial injury in COVID-19.

Therapeutic Approaches for Endothelial Injury in COVID-19	In Vitro Effects	In Vivo Effects
Inhaled NO	Has antiviral activty against SARS-CoV-2	Has pulmonary vasodilatation activity
Cilostazol	Binds effectively to SARS-CoV-2’s main protease and spike protein	Unstudied
Prostacyclin	Protects the endothelium and has anti-inflammatory effects	Improves endothelial damage repairing, has neoangiogenetic and antithrombotic activities

## Data Availability

Not applicable.

## Abbreviations

COVID-19	coronavirus disease 2019
SARS-CoV-2	severe acute respiratory syndrome coronavirus 2
ACE-2	angiotensin-converting enzyme 2
NO	nitric oxide
NOS	nitric oxide synthase
eNOS	endothelial NOS
ROS	reactive oxygen species
EDHF	endothelium-derived hyperpolarizing factor
Ang II	angiotensin II
AT1	angiotensin type 1
EV	extracellular vesicle
miRNA	micro RNA
SARS-CoV-1	severe acute respiratory syndrome coronavirus 1
MERS-CoV	Middle East respiratory syndrome coronavirus
IL	interleukin
TNF-α	tumor necrosis factor alpha
TF	tissue factor
vWF	von Willebrand factor
ICU	intensive care unit
PCR	Polymerase chain reaction
ACEI	angiotensin-converting enzyme inhibitor
ARB	angiotensin II receptor blocker
